# Tendon Repair Leads to better Long-Term Clinical Outcome than Debridement in Massive Rotator Cuff Tears

**DOI:** 10.2174/1874325001611010546

**Published:** 2017-07-25

**Authors:** Matthias Alexander König, Volker Alexander Braunstein

**Affiliations:** Department of Traumatology and Orthopaedic Surgery, Ludwig-Maximilians-University Munich, Munich, Bavaria, Germany

**Keywords:** Rotator cuff tear, Reconstruction, Debridement, Long-term follow-up, Outcome

## Abstract

**Introduction:**

Massive tears in the rotator cuff are debilitating pathologies normally associated with loss of function and pain. Tendon reconstruction is seen as the standard treatment in order to preserve shoulder function and to inhibit cuff associated osteoarthritis. However, the effect on longer-term shoulder function and patient satisfaction is unknown.

**Material and Methods:**

165 consecutive patients with massive tears were included. 57 debridement (mean age 61.9±8.7 years (range 43-77)) and 108 reconstruction (mean age 57.5±8.9 years (range 45-74)) cases could be followed up 2-4 (short-term), 5-6 (mid-term) and 8-10 (long-term) years after surgery. Evaluation was performed with the Constant, a modified ASES and the DASH score. Statistical analysis was done using Sigma-Stat Version 3.5 with a p-value<0.05 indicating statistical significant differences.

**Results:**

All three scoring systems showed no significant differences in the short-term follow-up for the two groups (mean values: Constant debridement/repair: 70±11.9/66±13.6; ASES debridement/repair: 22.3±3.3/ 23.3±3.3; DASH debridement/repair: 22.3±11.0/ 24.3±10.1). In the mid-term (Constant debridement/repair: 51±2.9/68.3±5.2; ASES debridement/repair: 20.3±1.3/24.3±1.7; DASH debridement/repair: 31.0±6.5/20.3±5.4) and long-term follow-up (Constant debridement/repair: 42.3±3.8 /60.7±2.6, ASES debridement/repair: 17.3±0.5/21.7±0.5, DASH debridement/repair: 41.3±6.2/25.0±1.4), rotator cuff reconstruction revealed better objective results and better patients’ satisfaction.

**Conclusion:**

Rotator cuff tendon repair leads to better long-term clinical outcome and subjective satisfaction compared to debridement. Tendon reconstruction should be considered as a treatment for patients suffering from massive rotator cuff tears, thus preserving shoulder function and by that means delay indication for reverse arthroplasty.

## INTODUCTION

As a four-part complex, the rotator cuff muscles and tendons enclose the whole humeral head allowing large rotational movement. Degenerative changes influence the tendons’ integrity, hence leading to imbalance of the shoulder joint and tendon tears [1]. Full thickness tears however can be absolutely asymptomatic with an incidence reported to be up to 54% in the population [2]. If the degenerative tear is very large, extended either anteriorly or posteriorly and involving two or more tendons, altered kinematics and pathological shear forces may lead to pain and to loss of shoulder function [1, 3]. Consequently, tendon degeneration is a subtle process leading to fatty muscle degeneration and tendon retraction. The loss of muscle integrity and tendon quality is making the treatment decision difficult for affected patients if compared to traumatic tears [4-7].

Direct tendon repair in either arthroscopic or open fashion is favourable for patients with high functional demands in professional and private life. The results of rotator cuff repair, however, are very inhomogeneous with a high re-rupture rate especially in the early post-operative period and persistent fatty muscle degeneration [8-15]. In addition, tendon repair has longer post-operative anastasis without the necessity of improvement in range of motion [16, 17].

Debridement procedures may offer an alternative to complex reconstructions with quicker pain relief as well as less post-operative in-hospital stay and rehabilitation [18-24]. Despite short surgery and post-operative care, functional results after debridement seem to be inferior to rotator cuff repair in short and mid-term follow-ups [25-27].

Therefore, the main goal of this study was to assess objective and subjective outcome after repair of the rotator cuff or debridement procedures for massive rotator cuff tears over a mid-term and long-term follow-up period [28].

## MATERIAL AND METHODS

### Study Design

A retrospective analysis of medical records was performed. The inclusion criteria of the study were a chronic tear with a tendon retraction of 4-5 centimetres [6, 10, 29-31]. Exclusion criteria included isolated subscapularis tear, traumatic tears or pre-operative infected shoulders. A MRI scan was performed prior to surgery in all patients to determine size and location of the rupture and its degree of tendon retraction. The selected patients had similar tear sizes and tendon retractions.

Over all, between 2004 and 2006, 165 consecutive patients could be identified and included in the study for review, 108 of those underwent rotator cuff reconstruction and 57 debridement.

The debridement group included 32 female and 25 male patients with a mean age of 61.9 ± 8.7 years (range 43 to 77 years). 4 isolated supraspinatus tears were identified, 35 had tendon ruptures in the supra- and infraspinatus muscle, leaving 6 patients with tears in subscapularis and supraspinatus tendons and 12 with involvement of three rotator cuff tendons.

34 female and 74 male patients with a mean age of 57.5 ± 8.9 years (range 45 to 74 years) were included in the reconstruction group. 33 patients had isolated supraspinatus tendon ruptures, 17 had involvement of the subscapularis and supraspinatus tendon and 44 tears in the supra- and infraspinatus tendon. 13 patients were treated for tears in three muscle tendons and one case of an isolated infraspinatus tear was included.

### Surgical Technique

All patients were treated in a single centre by two surgeons. The debridement procedure included arthroscopic subacromial decompression with acromioplasty and residual tendon debridement in beach chair position (3 os acromiale resections). The acromionizer burr and a full radius 5.5 mm resecter were used to carefully smoothen the tendon remnants and to trim the greater tuberosity according to Scheibel *et al*. [24] creating a satisfactory subacromial space. Post-operative physiotherapy was started the next day as well with passive motion exercise for 3 weeks and increased physical activity after that to increase muscle strength.

In comparison, the rotator cuff tendon reconstruction was performed as a mini-open procedure: After usual beach chair positioning a standard posterior portal was created and a diagnostic arthroscopy performed to confirm the location and extension of the tendon tear. Acromioplasty with a bursectomy followed through the lateral portal with a full radius resecter (5.5mm) and acromionizer burr in every patient. In two cases, resection of an os acromiale was necessary. After the subacromial decompression a skin incision was made above the tear assessing the rotator cuff through a deltoid split approach. A modified Mason-Allen tendon-grasping technique with Ethibond 1.0 was used in the affected tendons followed by trans-ossary re-insertion. Physiotherapy was started the day after surgery with passive motion exercises for three weeks and active exercises for a total of 3 months.

### Outcome Measurements

The Constant Score according to Boehm *et al.* [32, 33] and DASH Score [33] were used as objective outcome measurements. For the subjective outcome, the ASES score [34] was modified to focus on patients’ satisfaction with a possible maximum score of 30 indicating high satisfaction. The short-term follow-up was defined as 24 to 48 months, the mid-term follow-up 60 to 72 months and the long-term follow-up 96 to 120 months after surgery. The statistical analysis was performed with the Mann-Whitney Rank-Sum Test (SigmaStat Version 3.5) for non-metric parameters. A p-value of p < 0.05 was seen as statistical relevant difference.

No radiological follow-up was achieved due to the high re-tear rate reported after tendon repair.

## RESULTS

### Constant Score

**Fig. (1):**
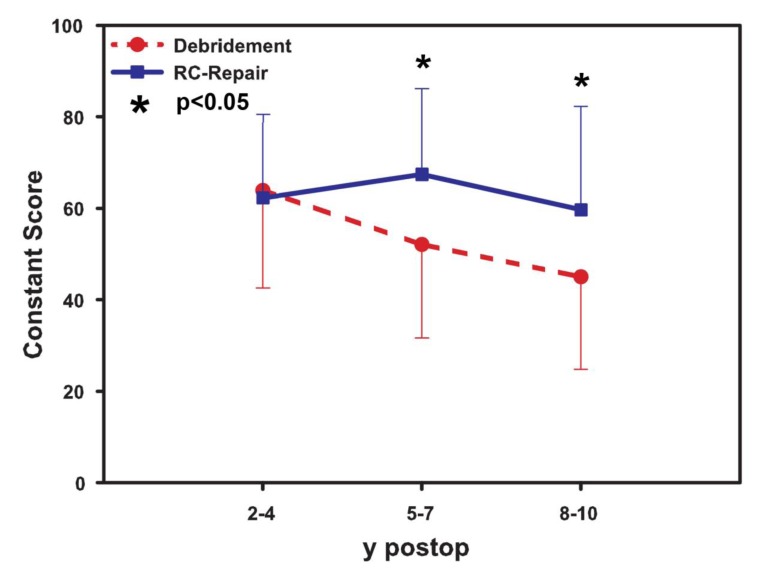
Results of the Constant score indicating significant changes in the mid-term and long-term follow up of rotator cuff tendon repair and debridement. No significant differences were found at short-term follow-up.

The mean Constant score (Fig. **1**) at the short-term follow-up of 24-48 months was slightly higher for the debridement group (mean value 70±11.9) compared to the reconstruction group (mean 66±13.6) (p>0.05).

At the mid-term follow up of 60-72 months, the rotator cuff repair group revealed better outcome compared to the debridement (mean value 68.3±5.2 *vs*. 51±2.9, p< 0.05). At the final long-term follow-up, the mean score in the rotator cuff tendon reconstruction group was 60.7±2.6 compared to 42.3±3.8 in the debridement group (p< 0.05).

### Modified ASES Score

**Fig. (2):**
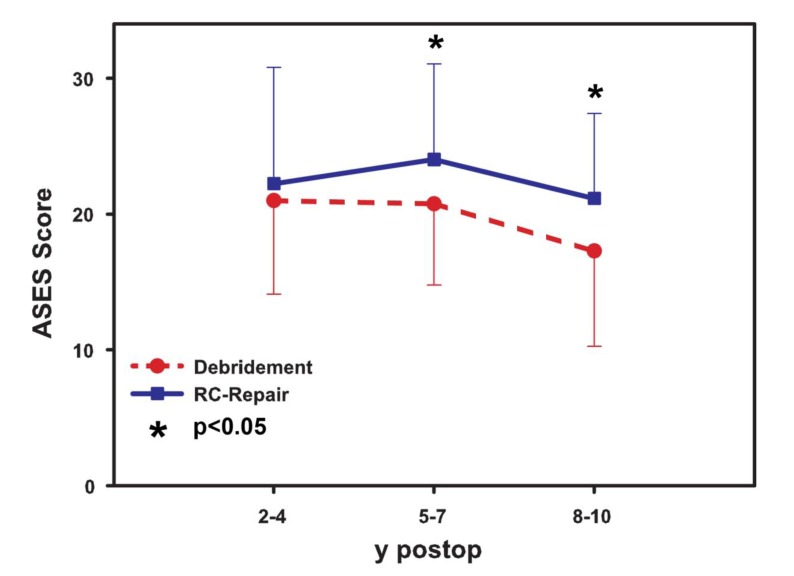
Results of the modified ASES score (Table **1**) for subjective outcome after tendon repair and debridement. Significant difference in mid-term and long-term follow-up is seen,whereas the short-term follow-up showed no statistically significant differences.

No significant statistical (Fig. **2**) difference was seen at the short-term follow-up (mean score 23.3±3.3 in the reconstruction *vs*. 22.3±3.3 in the debridement group, p> 0.05). However, a significant difference was seen in the mid-term follow-up (mean value 24.3±1.7 for the reconstructive surgery and 20.3±1.3 in the debridement group, p< 0.05) as well as in the long-term follow-up (mean score result 21.7±0.5 for tendon repair and 17.3±0.5 for the debridement, p< 0.05).

### DASH Score

**Fig. (3):**
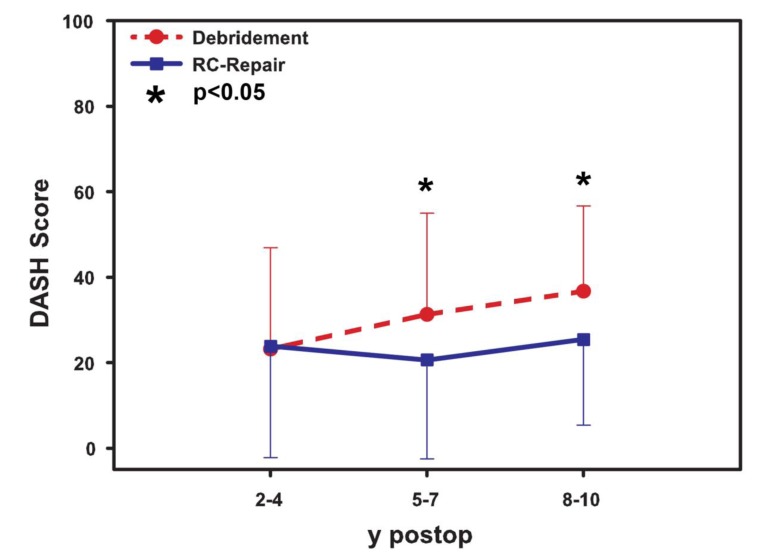
DASH scoring system with significant differences between cuff repair and debridement in mid-term and long-term follow-up.

In the DASH scoring (Fig. **3**) system, the tendon reconstruction showed no significant benefit in a short-term follow-up if compared to the debridement (mean value 24.3±10.1 to 22.3±11.0, p >0.05). The mean value in the mid-term follow-up was 20.3±5.4 for the tendon reconstruction and 31.0±6.5 for the patients treated with debridement (p< 0.05). In the long-term follow-up, the tendon repair showed statistically significant better results than the debridement group (mean 25.0±1.4 *vs*. mean 41.3±6.2, p< 0.05).

## DISCUSSION

This investigation is showing the results of debridement and tendon reconstruction surgery in mid-term and long-term follow-ups for massive rotator cuff tears. Interestingly, the debridement group had equal results in short term to the reconstruction group. Over the time, better results are achieved if patients had tendon reconstruction as seen in the Constant and DASH score results. Focussing on patients’ satisfaction, the modified ASES score showed better results in the repair group as well at mid-term and long-term follow-up. All tools used in this study seem to be reliable for further studies.

Treatment of massive rotator cuff tears is still challenging for surgeons and was discussed controversially in the past between reconstructive tendon repair techniques and palliative surgical options putting the focus mainly on pain relief. This could cause problems to determine the best treatment options for the individual.

Tendon repair was deemed to be the gold standard for young patients with high demands to shoulder function [9-17]. Reconstructive surgery is however correlated with longer surgery time and prolonged anastasis [16, 17], which might not be suitable for elderly patients. As massive tears are normally a result of degenerative changes, biomechanical properties of repaired tendons and cuff healing might be inferior due to the long time span between tear and repair [7, 15]. Structural analysis with post-operative MRI scans after reconstruction showed increased fatty infiltration with a re-tear rate of 57%, but excellent clinical outcomes in mid- and long-term follow up examinations [6, 10]. Arthroscopic repair of large and massive rotator cuff tears also led to excellent pain relief and shoulder function postoperatively, but re-tears were seen frequently with a significant deterioration of clinical outcomes after a follow-up of 2 years [12]. Even mini-open repair did not provide a watertight cuff repair, but satisfactory mid-term clinical results [9, 15].

Although good clinical results and pain relief were achieved, structural integrity after tendon repair seemed not very satisfactory, hence questioning the need of reconstructive surgery. Open or arthroscopic subacromial decompression and debridement were introduced as salvage options with less duration of surgery putting the focus on pain relief. Apoil *et al.* showed already in 1977 good pain relief and satisfactory shoulder function after open debridement [19]. If coronal (remaining inferior portion of the rotator cuff versus the deltoid muscle movement) and transversal plane (subscapularis versus infraspinatus and teres minor) balance could be maintained, normal shoulder function was achieved after tendon debridement, subacromial decompression and an adequate acromioplasty [22, 23].

Even though inferior objective results after debridement compared to tendon repair occurred directly after surgery, significant pain relief and improvement of shoulder function in terms of range of motion was achieved. Patients were able to perform daily life activities and the majority could return to work with occasional residual weakness in external rotation [20, 24, 27].

Interestingly, Motycka *et al.* showed no advantage of tendon reconstruction compared to debridement [28].

Unfortunately, the majority of the studies have no long-term follow-up results regarding clinical outcomes and patient satisfaction for both surgical options. This study indicates that tendon reconstruction even in massive rotator cuff tears offers better clinical outcomes with a high patient satisfaction in a long-term follow-up. Debridement should only be considered if short-term pain relief is the main goal. This is supported by the findings that in the short-term follow-up, no statistical significant differences were found in the scoring systems. Deterioration however was already found after the mid-term follow-up. Regarding the aging population, tendon repair could be the treatment method of choice for massive rotator cuff tears.

This study has several limitations. Due to the retrospective study design, pre-operative information regarding shoulder function was not available for all the patients included in this study, making a pre-operative status and statistical analysis impossible. Since complete data sets were collected during the first follow-up, this was defined as starting point of the investigation.

In addition, mini-open repair is no longer the gold standard for tendon reconstruction. Total arthroscopic repair gained popularity and importance in the past and is now deemed to be the treatment option of choice for tendon repair. This new technique might have even better outcome measurements even in short-term follow-up studies. It will still last however a couple of years until a similar follow-up period like in this study is reached and results are comparable. On the other hand, the results indicate that mini-open reconstruction seem to be a reliable treatment option.

A radiological follow-up was not performed in this study but as mentioned above, re-tear rates of up to 57% are reported in the literature [6, 10], which lead to the idea to focus on clinical outcomes and patient satisfaction. The issue of humeral head migration, cuff integrity and fatty degeneration could be topics of further investigations.

## CONCLUSION

Debridement is an easy to perform and short procedure and seems to be an option especially for the elderly. However, functional outcome and patient satisfaction is inferior to tendon repair techniques in a mid and long-term follow-up. This indicates that cuff reconstruction in large, degenerative tears can maintain a good shoulder function and postpone the need of reverse shoulder arthroplasty even in the elderly patient.

## Figures and Tables

**Table 1:** Modified ASES score for subjective outcome according to the original ASES score [36] and interpretation of results. The maximum score of 30 indicates a good outcome

ACTIVITY	RIGHT ARM	center ARM
1. Put on a coat	0 1 2 3	0 1 2 3
2. Sleep with your painful or affected side	0 1 2 3	0 1 2 3
3. Wash back/ so up bra in back	0 1 2 3	0 1 2 3
4. Manage toiletting	0 1 2 3	0 1 2 3
5. Comb hair	0 1 2 3	0 1 2 3
6. Reach a high shelf	0 1 2 3	0 1 2 3
7. Lift 10 lbs above shoulder	0 1 2 3	0 1 2 3
8. Throw a ball overhand	0 1 2 3	0 1 2 3
9. Do usual work	0 1 2 3	0 1 2 3
10. Do usual sport	0 1 2 3	0 1 2 3
30 – 25	Excellent
25 – 20	Very good
20 – 15	Good
15 – 10	Moderate
10 – 5	Poor
5 – 0	Very poor
